# Tamm-Horsfall Glycoprotein Enhances PMN Phagocytosis by Binding to Cell Surface-Expressed Lactoferrin and Cathepsin G That Activates MAP Kinase Pathway

**DOI:** 10.3390/molecules16032119

**Published:** 2011-03-03

**Authors:** Syue-Cian Siao, Ko-Jen Li, Song-Chou Hsieh, Cheng-Han Wu, Ming-Chi Lu, Chang-Youh Tsai, Chia-Li Yu

**Affiliations:** 1 Institute of Molecular Medicine, National Taiwan University College of Medicine, No. 7 Chung-Shan South Road, Taipei 100, Taiwan; 2 Department of Internal Medicine, National Taiwan University Hospital and National Taiwan University College of Medicine, No. 7 Chung-Shan South Road, Taipei 100, Taiwan; 3 Division of Allergy, Immunology and Rheumatology, Buddhist Dalin Tzu-Chi General Hospital, No. 2 Ming-Shen Road, Dalin, Chia-Yi, Taiwan; 4 Section of Allergy, Immunology and Rheumatology, Taipei-Veterans General Hospital, No. 201 Section 2, Shih-Pai Road, Taipei 11217, Taiwan

**Keywords:** Tamm-Horsfall glycoprotein, neutrophil phagocytosis-enhancing activity, lactoferrin, cathepsin G, protein-core structure, carbohydrate-side chain

## Abstract

The molecular basis of polymorphonuclear neutrophil (PMN) phagocytosis-enhancing activity (PEA) by human purified urinary Tamm-Horsfall glyco- protein (THP) has not been elucidated. In this study, we found human THP bound to lactoferrin (LF) and cathepsin G (CG) expressed on the surface of PMN, identified by a proteomic study with MALDI-TOF- LC/LC/mass spectrometric analysis. Pre-incubation of 10% SDS-PAGE electrophoresed PMN lysates with monoclonal anti-LF or anti-CG antibody reduced the binding with THP. To elucidate the signaling pathway of THP on PMN activation, we found THP enhanced ERK1/2 phosphorylation, reduced p38 MAP kinase phosphorylation, but had no effect on DNA binding of the five NF-κB family members in PMN. To further clarify whether the carbohydrate-side chains or protein-core structure in THP molecule is responsible for THP-PEA, THP was cleaved by different degrading enzymes with carbohydrate specificity (neuraminidase and β-galactosidase), protein specificity (V8 protease and proteinase K) or glycoconjugate specificity (carboxylpeptidase Y and O-sialoglycoprotein endopeptidase). We clearly demonstrated that the intact protein-core structure in THP molecule was more important for THP-PEA than carbohydrate-side chains. Putting these results together, we conclude that THP adheres to surface-expressed LF and CG on PMN and transduces signaling via the MAP kinase pathway to enhance PMN phagocytosis.

## 1. Introduction

Tamm-Horsfall glycoprotein (THP), a renally excreted 80–90 kDa GPI-anchored macromolecule, is an important defense molecule in protecting urinary tract epithelial cells from microbial invasion [1,2]. This macromolecule contains approximately 25–35% of florid carbohydrate-side chain structure with abundant sialic acid [3,4]. Functionally, THP is capable of binding with diverse soluble proteins including complement components C1 and C1q [5,6], immunoglobulin light chains [7], human serum albumin [8], interleukin 1 [9], interleukin 2 [10], and tumor necrosis factor-α [11]. In addition, THP can bind to the surface membrane of polymorphonuclear neutrophils (PMN) [12], lymphocytes [13], monocytes/macrophages [14] and renal glomerular mesangial cells [13] as a non-specific binder to affect cell functions. In our previous reports, we found THP bound to surface-expressed 32 kDa and 60 kDa molecules on PMN to enhance phagocytosis [15]. However, the nature of the molecule(s) expressed on PMN surface capable of reacting with THP and the molecular basis of THP-induced PMN phagocytosis-enhancing activity (THP-PEA) remain unelucidated. Mo *et al.* [16] demonstrated that the mannosylated-THP could bind to uroplakin Ia receptors expressed on the urothelial surface to prevent type I-fimbriated *E-coli* adhesion to urinary epithelial cells. Pfistershammer *et al.* [17] found that scavenger receptors, SREC-1, Cla-1 (SR-B1), and SR-A1 on dendritic cells were the cellular receptors for Tamm-Horsfall protein. Saemann *et al.* [18] reported that THP bound to TLR4 that was the molecule responsible for linking innate immune cell activation with adaptive immunity. 

It is conceivable that Tamm-Horsfall glycoprotein is exclusively synthesized by the renal tubular cells in the thick ascending limb of Henle’s loop [19]. In a pathological sense, mutation within the THP gene involves familial juvenile hyperuricemic nephropathy, glomerulocystic kidney disease and autosomal dominant medullary cystic kidney disease type 2 [20,21,22]. Ablation of THP gene increases susceptibility of mice to bladder colonization by type 1-fimbriated *Escherichia coli* [16]. In our previous study, we found THP purified from normal human urine exhibited immuno-modulatory effects on lymphocytes [23], monocytes [23], PMN [15] and renal glomerular mesangial cells [13] via binding with surface expressed 60 kDa and 32 kDa molecules. However, inhibition tests revealed that pre-incubation of a number of monosaccharides abundant in the carbohydrate-side chains of THP molecules including *N*-acetylneuraminic acid, *N*-acetylgalactosamine, *N*-acetylglucosamine and α-methyl-D-mannoside failed to inhibit the mitogenic effect of THP on human mononuclear cells [13]. These results suggest that THP is capable of reacting with surface expressed molecules on different cells, but not through specific CHO containing lectin-like receptors on the cells. In the present study, we aimed to identify the nature of the binding molecules of THP on PMN surface and elucidate the signaling pathway(s) in THP-PEA. 

## 2. Results and Discussion

### 2.1. PMN phagocytosis-enhancing activity of THP is initiated by binding to PMN surface that depolarizes membrane potentials

**Figure 1:**
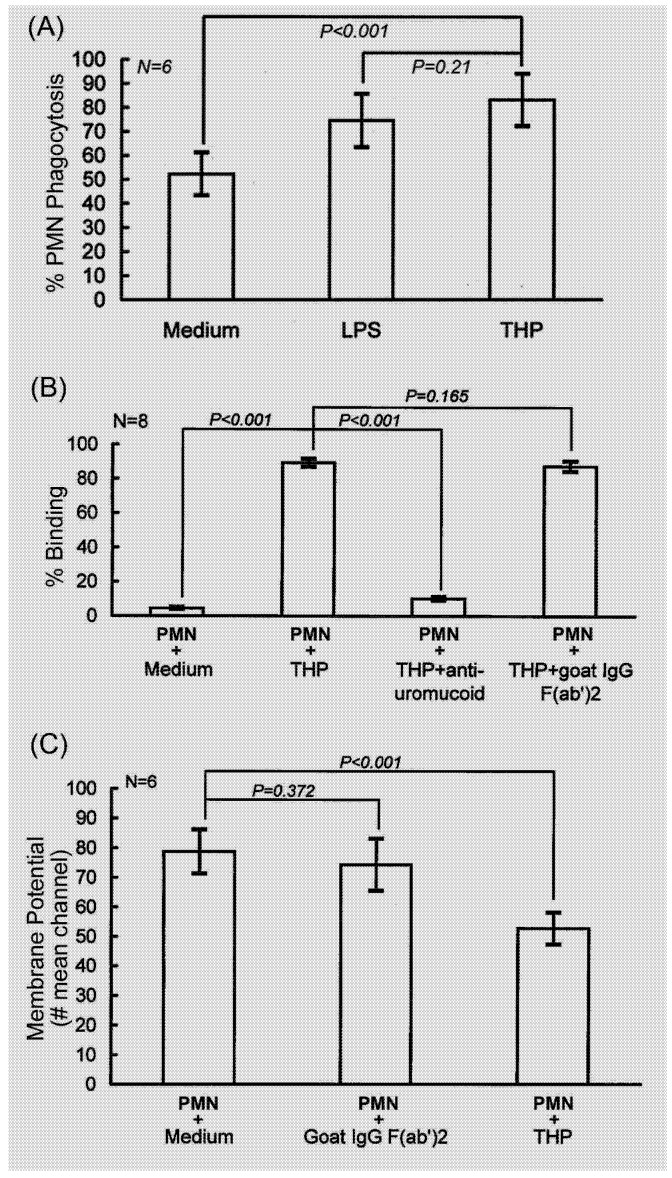
Effect of THP on phagocytosis and membrane potential changes of PMNs. (A) THP (25 μg/mL) enhances PMN phagocytosis detected by flow cytometry. (B) Pre-incubation of THP with anti-THP (anti-uromucoid) antibodies reduces THP binding with PMN detected by flow cytometry. (C) THP decreases resting membrane potentials of PMN after incubation for 30 min.

First, we confirmed again that THP (25 μg/mL) significantly enhanced PMN phagocytosis even higher than LPS (100 μg/mL), as shown in Figure 1(A). 

Pre-incubation of THP with anti-uromucoid antibodies suppressed THP-induced PEA as shown in our previous study [15]. The THP-induced PEA was through THP binding on the PMN surface [Figure 1(B) that subsequently depolarized membrane potentials of PMN [Figure 1(C)]. In contrast, goat non-specific IgG-F(ab^′^)_2_ did not affect membrane potentials of PMN. These results suggest that THP could activate PMN to enhance PMN phagocytosis via its surface binding activity.

### 2.2. Identification of the binding molecule(s) of THP on PMN surface

**Figure 2:**
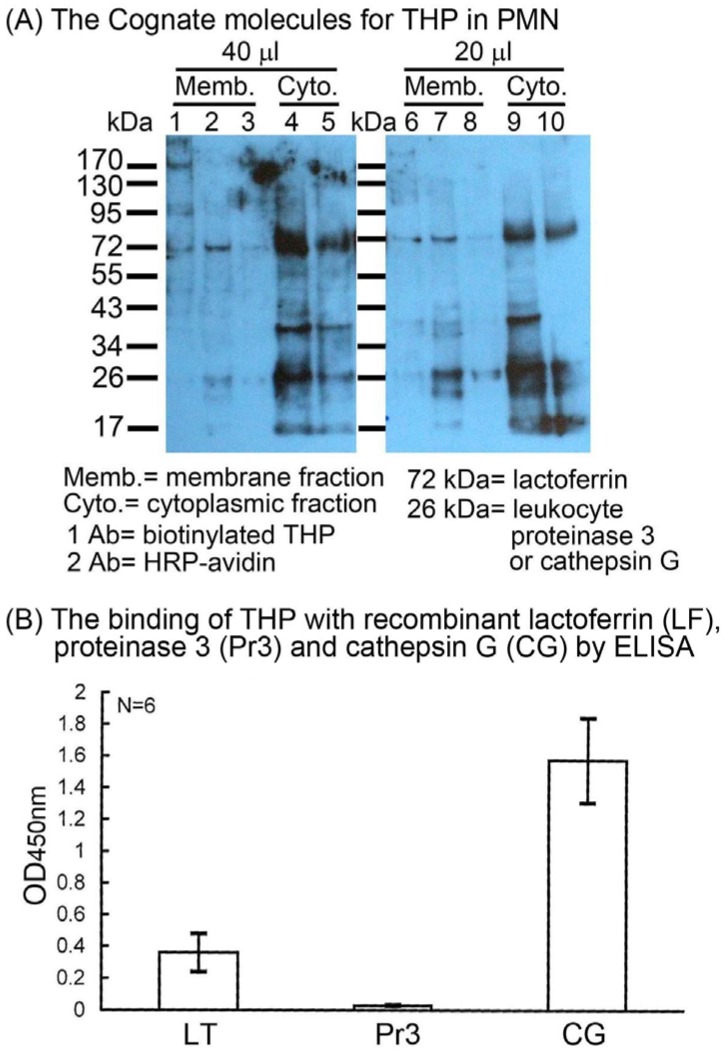
Identification of the surface-expressed molecules on PMN that binds with THP was detected after 10% SDS-PADE (A) and ELISA (B). (A) The membranous and cytosolic molecules were extracted from PMN (1 × 10^7^ cells/mL) by commercially available Active Membrane Protein Extraction kit. Membrane proteins (Memb. 40λ in lances 1-3 and 20λ in lanes 9–10) and cytosolic proteins (Cyto. 40λ in lances 4–5 and 20λ in lanes 9–10) of PMN were electrophoresed in 10% SDS-PAGE. Then, the molecules were reacted with biotinylated THP and HRP-conjugated streptavidin. Two bands (72 kDa and 26 kDa) were constantly present after reacting with THP. (B) The binding capacity of biotinylated THP with recombinant human milk lactoferrin (72 kDa), human neutrophil proteinase 3 (26 kDa) or cathepsin G (26 kDa) pre-coated in the microwells was detected by ELISA.

We next tried to identify the nature of the binding molecule(s) on PMN surface capable of reacting with THP. We isolated the membranous and cytosolic molecules from PMN by a commercially available native membrane protein extraction kit. The extracted membranous and cytosolic molecules were electrophoresed in 10% SDS-PAGE and then probed by biotinylated THP. We noted that the 72 kDa and 26 kDa molecules were constantly present in membranous and cytosolic extracts when probed by biotinylated THP [Figure 2(A)]. 

**Figure 3:**
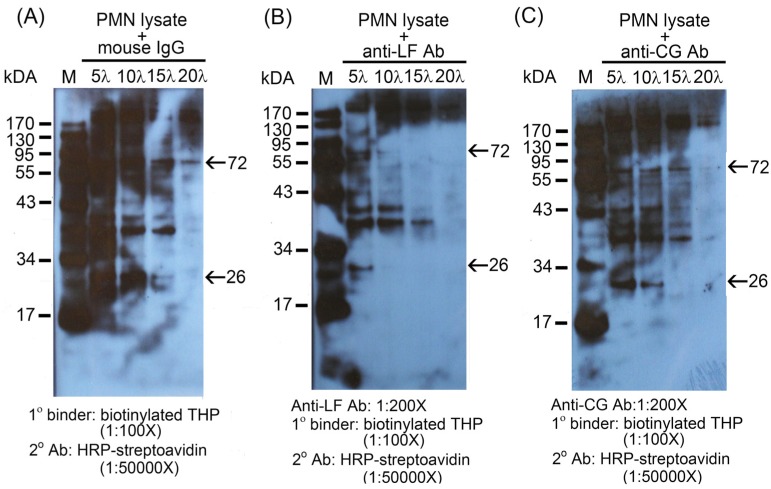
Pre-incubation of anti-lactoferrin (anti-LF), anti-cathepsin G (anti-CG), or mouse non-specific antibody with PMN lysates block the reaction between biotinylated-THP with PMN lysates. Different amounts (5~20 λ) of mouse non-specific IgG (A), anti-LF antibody (B), and anti-CG antibody (C) preincubation with 10% SDS-PAGE electrophoresed total PMN lysates (1 × 10^7 ^cells/mL) before probed by biotinylated THP.

Proteomic study with MALDI-TOF-LC/LC/mass spectrometric analysis revealed that the 72 kDa molecule was highly homologous with human milk lactoferrin (LF) whereas the 26 kDa molecule is homologous with human neutrophilic proteinase 3 (Pr3) or cathepsin G (CG). To further confirm these three proteins are really the binding molecules of THP expressed on PMN surface, recombinant human LF, Pr3, and CG were coated on microwells and the binding capacity with biotinylated-THP was measured by ELISA. As shown in Figure 2(B), LF and CG, but not Pr3, were reactive with THP. Moonen *et al.* [24] demonstrated that THP bound strongly to denatured TNF-α when the molecules were fixed to the microwells. However, we detected the binding between THP and microwell-bound different proteins including BSA, human IgGs, C1q, TNF-α, IL-6, IL-8 by ELISA [8], and different viable cells including PMNs, RBCs, and rat glomerular mesangial cells by flow cytometry [13]. We conclude that THP is a non-specific binder capable of binding with both natural and denatured protein molecules. Next, the inhibition tests were conducted that the total PMN lysates electrophoresed in 10% SDS-PAGE were pre-incubated with mouse non-specific IgG [Figure 3(A)], antibody against LF [Figure 3(B)] or CG [Figure 3(C)]. 

The complexes were probed by biotinylated-THP. We noted that smudges were prominent in non-specific mouse IgGs staining as expected since a lot of antibodies against different environmental immunogens were contained in the mouse IgGs reservoirs. However, the density of many bands (such as 110 kDa, 50 kDa, 40 kDa and 37 kDa molecules) did not underlying big change in Figure 3 panels A, B and C. Panel B was conducted by pre-incubation with monoclonal antibody against lactoferrin. Both 72 kDa and 26 kDa bands declined in parallel with increasing quantity of anti-LF. This may suggest certain common epitopes exist between LF and CG recognized by anti-LF. In contrast, anti-CG pre-incubation (panel C) declined both 26 kDa and 72 kDa bands in higher amount (20 λ) of anti-CG. The other bands seemed not be affected much. Although the actual cause for this non-specific inhibition by a rather small amount of antibodies remains unclear, we deduce that cross-reactivity of anti-LF against LF and CG is greater than anti-CG against CG and LF. 

Lactoferrin is an iron-containing protein usually found in the secondary granules of PMN and is released after activation [25]. Interestingly, this molecule may also translocate to the surface of PMN spontaneously, even in non-activated PMN [26]. In contrast, other neutrophil granular proteins such as cathepsin G, elastase, myeloperoxidase, proteinase 3, and tumor necrosis factor-α translocate to the cell surface only on activation [27,28,29,30,31]. These secondary granules of PMN were considered initially as reservoirs of proteolytic enzymes for defense purposes. Recent evidence disclosed that the secondary granular membrane can fuse with the surface membrane after activation and serves as new receptors or ligands in response to the environmental modalities [31,32,33,34]. Accordingly, it is quite possible that the surface membrane-expressed LF and CG may serve as a THP receptor to enhance PMN phagocytosis via the MAPK signaling pathway. Although a proteomic study revealed that the 26 kDa protein molecule was homologous with either CG or Pr3, the binding capacity between Pr3 and THP by ELISA was extremely low [Figure 2(B)]. We may tentatively neglect Pr3 as a binding molecule of THP. The antibody blocking experiments also suggest LF and CG are the binding molecules for THP [Figure 3(B) and Figure 3 (C)]. Obviously, our results were different from those of other authors. Saemann *et al.* [18] by using activated myeloid dendritic cells (DCs) as targets demonstrated that THP triggered typical TLR signaling via TLR4. Pfistershammer *et al.* [17] identified scavenger receptors SREC-1 expressed on endothelial cells as THP binding molecule by using a retroviral expression cloning method. But the other two scavenger receptors (SR-B1 and SR-A1) on DCs exhibited low affinity with THP. THP is regarded as a broad non-specific binder for surface expressed proteins on different blood cells and glomerular mesangial cells, as reported in our previous study [13]. It is possible that different target cells used in different THP binding assay involved different binding molecules. We deduced that THP bound to surface-expressed LF and CG on PMN and then stimulated expression of complement receptor type 1 and type 3 on PMNs leading to PEA, as reported in our previous study [15]. We believe that THP receptors that may express on the different types of cells are multiple. 

### 2.3. THP-induced phosphorylation of MAP kinase signaling pathways in PMN

**Figure 4:**
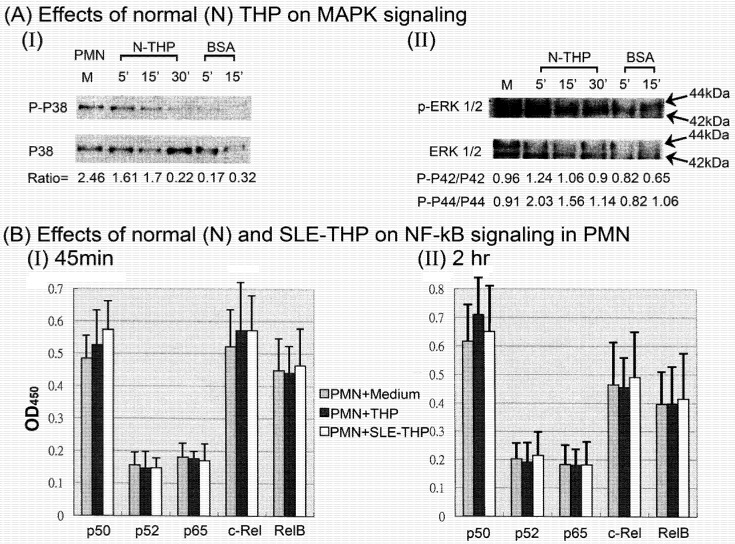
Effects of normal (N-THP) and SLE-THP on p38 and ERK1/2 MAP kinase phosphorylation (A) and NF-κB family member DNA binding capacity (B) in PMN. (A) N-THP and SLE-THP decreased phosphorylated-p38/P38 ratio in 5–30 min incubation (I) but increased phosphorylated-ERK1/2/ERK1/2 radio of PMN after 5’~15’ incubation (II). (B) Effects of 3 N-THP and 3 SLE-THP on DNA binding activity of 5 NF-κB family members in PMN after 45 min (I) and 2 h (II) incubation. No statistical difference was found among the three groups.

To further explore the signaling pathway of THP-mediated PEA, the expression and phosphorylation of MAP kinase signaling molecules in THP-activated PMN were investigated. It is quite interesting that the baseline ratio of phospho-p38 (p-p38)/p38 (=2.46), phospho-p42/p42 (=0.96), and phospho-p44/p44 (=0.92) in PMN incubation with medium were relatively high. The possible explanations for the increased baseline in PMN signaling are: (1) resting PMNs are not really in the rest status since PMNs as the first line defense cells are prepared ready for rapid response; (2) the isolation procedures, including dextran sedimentation, Ficoll-Hypaque density gradient centrifugation, incubation in chilled 0.83% ammonium chloride lysis solution, and several washes easily activate PMN. We found THP suppressed p-p38/p38 ratio gradually from 5 to 30 min [Figure 4(A)-I] whereas the phospho-ERK^1^/_2_/ERK^1^/_2 _ratio including p42 and p44 isoforms were enhanced in 5 min and gradually declined from 15 to 30 min [Figure 4(A)-II]. Interestingly, BSA remarkably suppressed both p38 and ERK1/2 phosphorylation. BSA is commonly used as the stabilizer for drugs or protein molecules in suspension. It is also possible that BSA can stabilize the intracellular signaling of the constantly active cells such as PMNs. In contrast, the DNA-binding activity of five family members of NF-κB including p50, p52, p65, c-Rel and RelB was not affected by THP derived from three normal controls and three patients with SLE in 45 min [Figure 4(B)-I] or 120 min incubation [Figure 4(B)-II]. In these experiments, SLE-THP was merely used as a disease control for normal THP. These results indicate that one of the signaling pathways of THP-induced PEA is through MAPK. However, the detailed signals involving THP-induced PEA is now under investigation.

### 2.4. Comparison of intact THP molecule and its enzyme-digested products on PMN phagocytosis

**Figure 5:**
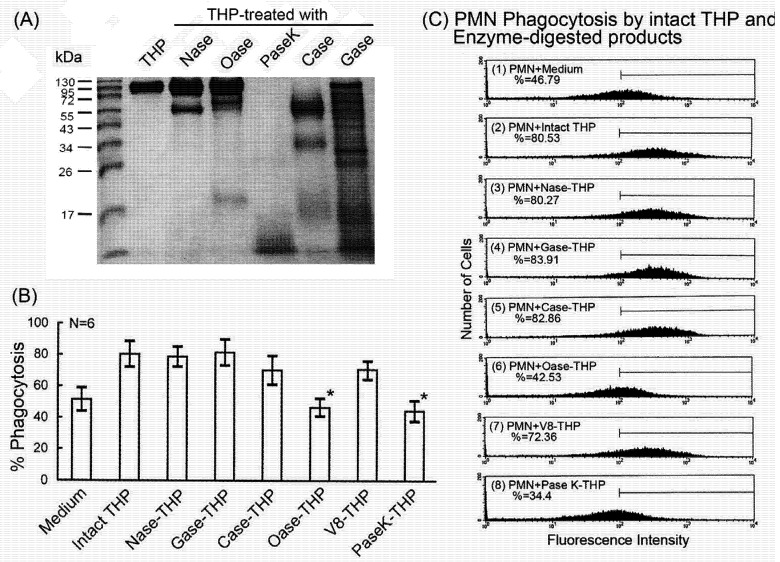
Comparison of PMN phagocytosis-enhancing activity of intact THP and its enzyme-cleaved products. (A) THP was digested by carbohydrate-degrading enzymes [neuraminidase (Nase), and β-galactosidase (Gase)], glycoconjugate-degrading enzymes [carboxypeptidase Y (Case) and O-sialoglycoprotein endopetidase (Oase)] and proteolytic- enzymes [V8 protease (V8) and proteinase K (PaseK)]. The intact THP molecule and its enzyme-cleaved products were then electrophoresed in 10% SDS-PAGE followed by Coomasie blue stain. (B) The effects of intact THP molecule and its different enzyme-cleaved products on PMN phagocytosis. (C) A typical case of PMN phagocytosis mediated by intact THP molecule and its enzyme-degrading products. The same experiments were performed three times with a similar tendency.

THP arbitrarily contains 25–35% complex carbohydrate-side chains in the molecule. It is believed that the carbohydrate-side chains mediate the diverse biological activities of THP as suggested by many authors [9,10,11,12]. However, only a few reports have discussed the biological functions of the protein-core structure in the THP molecule. Huang *et al.* [7] investigated the monoclonal IgG light chain-binding domain in THP molecule by deglycosylation and limited trypsin digestion. The authors found a band of 24.6 kDa peptide located between 6^th^ and 287^th^ amino acid residue of THP mediated light chain-THP interaction. This result is consistent with the fact that the protein-core in the THP molecule is important for protein-protein interactions, as seen in our study [8]. In the present study, we attempted to determine the contribution of CHO-side chains and protein-core structure of THP in enhancing PMN phagocytosis. Accordingly, THP was digested by carbohydrate-specific (Nase and Gase), protein-specific (PaseK and V8) or glycoconjugate-specific (Oase and Case) enzymes to deplete specific carbohydrate moieties or cleave the protein-core structure [Figure 5(A]. The phagocytosis-enhancing activity of intact and enzyme-digested THP products was then compared [Figure 5(B)]. 

We found that the specific carbohydrate moiety depletion by Nase and Gase did not affect THP-PEA. In contrast, intact protein-core structure of THP was crucial for mediating PEA since Oase and PaseK digestion remarkably suppressed PEA [Figure 5(B)]. For avoiding the interference by enzymes *per se* on PMN phagocytosis, the enzyme-treated THP specimens were either heat-inactivated at 65 °C for 30 min or the individual heat-inactivated enzyme was added to THP-stimulated PMNs as enzyme control. A representative case is presented in Figure 5(C). It is believed that the glycomoiety structure of THP consists mainly of N-linked glycans with di-, tri-, and tetra-antennary types [35,36] and one N-glycosylation site with high mannose sequences [37]. The high mannose glycans of human THP are carried by Asn251 [38], Man6GlcNac2 and Man5GluaC2 [37] that can interact with type І fimbriated *Escherichia coli* [39]. The findings of the present study seem inconsistent with other reports that the carbohydrate-side chains mediate the major biological functions of THP [9,10,11,12,40,41]. Parsons *et al.* [42] further showed the role of sialic acid in cytoprotective activity of THP in urinary system against cytotoxic effects by toxic urinary cations. In contrast, a number of monosacchrides such as *N*-actylneuraminic acid, *N*-actylgalactosamine, *N*-acetylglucosamine, and α-methyl-D-mannoside those are abundant in THP carbohydrate-side chain structure [8] failed to inhibit THP activities in our previous study [13]. However, these results are quite compatible with our previous reports that intact protein-core structure is crucial for enhancing mononuclear cells and PMN functions [8]. 

## 3. Experimental

### 3.1. Reagents and antibodies

Bovine serum albumin (BSA), neuraminidase (Nase), β-galactosidase (Gase), proteinase K (PaseK) and carboxylpeptidase Y (Case) were purchased from Sigma-Aldrich Immunochemical Corp. (St. Louis, MO, USA). O-sialoglycoprotein endopeptidase (Oase) was obtained from Cedar Lane Laboratories Ltd. (Burlington, NC, USA). V8 protease (V8) was obtained from Merck-Calbiochem (San Diego, CA, USA). Recombinant human neutrophil proteinase 3 (Pr3) and cathepsin G (CG) were obtained from Athens Res & Tech, Inc. (Athens, Georgia, USA). Human milk lactoferrin (LF) and lipopolysaccharide (LPS, *E-coli* serotype 026:B6) were purchased from Sigma-Aldrich. Mouse monoclonal anti-LF antibody was obtained from Biodesign Inc. (Saco, ME, USA). Mouse monoclonal anti-human CG antibody was obtained from Santa Cruz Biotechnology Inc. (Santa Cruz, CA, USA). Mouse monoclonal antibody against p38, phospho-p38, ERK1/2 and phospho- ERK1/2 MAP kinase were purchased from Cell Signaling Technology, Inc. (Denver, MA, USA). HRP-conjugatedstreptavidin was purchased from Biolegend, Inc. (San Diego, CA, USA). NF-κB ELISA kit was purchased from Active Motif. Inc. (Carlsbad, CA, USA).

### 3.2. Purification of THP from urine of normal human individuals and patients with systemic lupus erythematosus (SLE)

Twenty-four hour urine was collected from normal individuals and three patients with SLE in clean glass bottles. The purification of THP was carried out as described by Hunt and McGiven [43]. After three cycles of 0.58 M NaCl precipitation, alkaline distilled water (pH 9.0 was adjusted by adding 1N NaOH) dissolution, and centrifugal removal of insoluble substance, the purity and relative molecular weight of the obtained mucoproteins were detected by 10% SDS-PAGE. For fear of bacterial endotoxin (LPS) contamination during THP purification, we took two processes to confirm the eligibility of THP specimens: (a) measurement of the LPS levels (<0.1 EU/mL) in the specimens by Limulus amoebocyte coagulation test obtained from commercially available kit (Sigma-Aldrich). (b) The addition of polymyxin B (10 μg/mL) to inactivate the LPS activity in the specimens. The identification of THP molecule was confirmed by Western blot after probing by anti-uromucoid antibody (The Binding Site Ltd, University of Birmingham Research Institute, Birmingham, UK). All of the urine and blood donors signed the informed consent forms approved by the Institutional Review Board and Medical Ethics Committee, National Taiwan University Hospital, Taipei, Taiwan.

### 3.3. Biotinylation of THP

One milliliter of THP at a concentration of 1 mg/mL was incubated with excess amounts of biotin-labeling buffer (0.3 mg biotin/mL in PBS, pH 7.2) (Boehringer-Mannheim Biochemicals, GmbH, Mannheim, Germany) at 4 °C for 1.5 h with continuous gentle shaking as in our previous study [44]. After centrifugation at 11,800×g for 15 min, the clear supernatant was dialyzed against alkaline distilled water, pH 9.0 to remove the free biotin. The preparations were then digested by carbohydrate-, glycoprotein-, and protein-specific degrading enzymes following the method reported by Sherblom *et al.* [8]. All incubations were performed at 37 °C for 16 h. The concentration of respective enzyme was neuraminidase (10 units/mL) in 50 mM sodium acetate, pH 5.0; β-galactosidase (0.05 units/mL) in 50 mM sodium acetate, pH 5.0; proteinase K (0.5 μg/mL) in 10 mM Tris, pH 7.5 with 1 mM MgCl_2_; V8 protease (1 μg/mL) in 50 mM phosphate buffer, pH 7.8 twice-treatment; carboxy-peptidase Y, (enzyme:substrate = 1:10) in 0.2 M pyridine-acetate buffer, pH 5.6; *O*-sialoglycoprotein endopeptidase (50 μg/mL) in PBS, pH 7.2. The enzyme-digested products of THP were intensively dialyzed against alkaline distilled water, pH 9.0 for 24 h with frequent changes of dialysate to remove the digested products less than 10 kDa. These digested THP products were then heated at 65 °C for 60 min to inactivate the residual enzymes in the mixture. The products were lyophilized and stored at −20 °C until used. In addition, the same concentration of individual heat-inactivated enzyme was added in THP solution to minimize interference from enzymes *per se* in PMN phagocytosis assays.

### 3.4. Isolation of PMN from human peripheral blood

Heparinized venous blood obtained from normal individuals was mixed with one-quarter volume of 2% dextran solution (molecular weight 464,000 Da) and incubated at 37 °C for 20 min. The leukocyte-enriched supernatant was collected and diluted with the same volume of Hanks' balanced salt solution. The supernatant was than layered on Ficoll-Hypaque density gradient solution (specific gravity 1.077) and centrifuged at 300×g for 30 min. The PMN was collected from the bottom. The residual red blood cells in PMN suspension were lysed by incubating the cell pellet in pre-chilled 0.85% ammonium chloride solution at 4 °C for 10 min. The purity and viability of PMN was greater than 90% confirmed by Wright's stain and trypan blue dye exclusion. The cell concentration was adjusted to 1 × 10^6^/mL in 10% fetal bovine serum (FBS) in RPMI-1640 (10% FBS-RPMI). The cell lysates were prepared by dissolving the cells in cell lysing buffer solution containing 50 mM borate, 150 mM NaCl, 1% NP-40, 0.5% sodium deoxycholate, and 25 mM phenylmethylsulphonyl fluoride, pH 8.0.

### 3.5. Detectionof PMN phagocytosis by flow cytometry

We followed the method reported by Shalaby *et al.* [45]. Briefly, PMN (1 × 10^6 ^cells/mL) were pre-incubated with THP (25 μg/mL) or 10% FBS-RPMI for 45 min. Commercial available fluoresbrit carboxylate microsphere (0.75 μm in diameter, Polysciences Inc. Washington, PA, USA) previously opsonized with fresh normal human serum at 37 °C for 60 min. were then added to the PMN suspension with a ratio of PMN: bead = 1: 100. The mixture was incubated at 37 °C for another 60 min, followed by centrifugation at 300 g for 30 min. to remove the free beads. The cells were then fixed in 2.5% paraformaldehyde for 10 min. Both percentage (%) and mean fluorescence intensity (MFI#, denoted by the mean channel number) were detected by FACsort flow cytometry (Becton-Dickinson, Mountain View, CA, USA) with 488 nm excitation.

### 3.6. Detection of the binding between THG and PMN by flow cytometry

PMN suspension (0.5 mL, 2 × 10^6 ^cells/mL) was incubated with 0.5 mL of 10% FBS-RPMI or THP (50 μg/mL) at 37 °C in 5% CO_2_-95% air for 60 min. After three washes, the cells were stained with FITC-conjugated anti-human uromucoid antibodies (The Binding Site Ltd.). The percentage (%) and mean fluorescence intensity (MFI#) were detected by FACSort flow cytometry with 488 nm excitation.

### 3.7. Estimation of membrane potentials of individual cell by flow cytometry

We followed the method described by Shapiro *et al.* [46]. The indicator dye for detecting membrane potential of the cells was 3, 3'-dihexyloxacarbocyanine iodide (Eastman Kodak, Rochester, NY, USA). The membrane potential changes of PMN were measured after incubation with THP (25 μg/mL) for 30 min by flow cytometry with 488 nm excitation.

### 3.8. Extraction of membranous and cytosolic proteins from PMN

Commercially available Native Membrane Protein Extraction Kit (ProteoExtract, Calbiochem, San Diego, CA, USA) was used to extract PMN membranous and cytosolic proteins according to the manufacturer's instructions. The extracted membrane proteins remained in non-denatured state suitable for proteomic study. The precipitated pellets were sonicated and used as the cytosolic proteins of PMN.

### 3.9. Detection of THP-binding molecules on surface membrane and cytosolic molecules of PMN after 10% SDS-PAGE analysis

Twenty and forty microliters of membranous and cytosolic proteins (200 μg/mL) were electrophoresed in 10% SDS-PAGE. After membrane transfer, the separated molecules were probed by biotinylated THP and HRP conjugated-streptavidin. The complexes were then color-developed and detected by the enhanced chemiluminescence (ECL) protein detection system (Amersham International, Amersham, UK).

### 3.10. Measurement of binding capacity between LF, Pr3 or CG and biotinylated THP by ELISA

Human recombinant lactoferrin, proteinase 3, or cathepsin G (10 μg/mL, 100 μL) dissolved in pH 9.7 Tris buffer were coated in microwells overnight at 4 °C. After several washes, the non-specific binding was blocked by incubation with 1% BSA solution. Then, biotin-conjugated THP (10 μg/mL, 100 μL) were added and incubated at 37 °C for 2 h. After several washes, the binding of THP with the three proteins was measured by ELISA reader (Dynex) at OD_ 450mm_ absorbance.

### 3.11. Detection of p38 and ERK1/2 MAP kinase phosphorylation in PMN lysates by Western blot

PMN (5 × 10^6^ cells/mL) were pre-incubated with THP (50 μg/mL) or BSA (50 μg/mL) for 5–15 min. followed by cell lysis. The quantity of p38 and ERK1/2 MAP kinase and their phosphorylated molecules were detected by Western blot probed by the respective antibody.

### 3.12. Detection of DNA binding activity of the five NF-κB family members in PMN nuclear extracts after incubation with THP

We used the Trans AM^TM ^NF-κB family transcription factor assay kit (Active Motif, Tokyo, Japan) to prepare the nuclear extract from normal human PMN (5 × 10^6^ cells/mL). The DNA binding activity of the 5 NF-κB family members with epitopes on p50, p52, p65, c-Rel and RelB were detected by ELISA. The detailed procedures were described in the manufacturer's instruction booklet. We incubated PMN with medium or THP for 45 and 120 min. The range of detection was from 0.2–0.5 μg of nuclear extract or 1–40 ng of recombinant p50 or p65 protein/well.

### 3.13. Statistical analysis

The results are represented by mean ± s.d. in the entire study. Continuous variables were assessed by non-parametric Wilcoxon rank-sum test using a commercially available software package: stata/SE8.0 for Windows. A *p* < 0.05 was considered significance.

## 4. Conclusions

Four original findings were derived from the present study: (1) LF and CG are the binding molecules of THP expressed on PMN surface; (2) THP transduces PMN activating signals via enhanced ERK ½, but suppressed p38 MAP kinase phosphorylation; (3) THP does not affect the DNA binding activity of the five NF-kB family members; (4) intact protein-core structure rather than carbohydrate-side chains in the THP molecule is crucial for PMN phagocytosis-enhancing activity. These findings indicate that human urinary THP bound to surface membrane-expressed lactoferrin and cathepsin G that enhances PMN phagocytosis via modulating MAP kinase phosphorylation. We also confirmed that THP-induced PEA is mainly mediated by intact protein-structure rather than CHO-side chains in THP molecule. The detailed domain structure in THP molecule to mediate different functions on different cell types is now under investigation.
